# History of a Case of Verminous Disease

**Published:** 1829

**Authors:** Benjamin S. Brown

**Affiliations:** Logan County, Ohio


Art. 5.—*History of a case of Verminous Disease.* By Benjamin S. Brown, M. D. *of Logan County, Ohio.*

On Sunday evening, the 29th of March, 1829, I was called to see the infant son of Mr. I. C. aged about 4 years. I was informed by the parents, that it had been suddenly attacked a day or two previous, with severe griping pains of the bowels, resembling spasmodic colic; that its agony was very soon so great, that they apprehended it would go into fits; that they gave it a tea-spoon-ful of spirit of turpentine, which afforded almost instantaneous relief; that after a short time they administered a dose of castor oil, which produced a few motions from its bowels, and brought away a few worms, (*lumbricoides.*) Upon examination, I found the abdomen much tumefied and very tender on pressure; several hard lumps or knots, could be distinctly felt in many parts, particularly along the course of the arch of the colon, and near the umbilicus; which regions especially were sore and painful on pressure. The pulse was about 60 in a minute, quick, somewhat depressed, but regular. The tongue had rather a white, smooth, slimy appearance; breath of a peculiar, disagreeable odour; the breathing was nearly natural; had no appetite since taking the castor oil, though I was told it had been very voracious for some months before, causing it to eat as much as was usual for two or three children of its age. The complexion was pale and sallow, with an anxious unmeaning expression of countenance; the lips appeared to be swelled, particularly the upper one.

It being late in the evening, I gave a portion of calomel, about 8 grains, combined with a carminative. On the morning of the 30th, no operation of the calomel having taking place, I made a decoction of spigelia and senna, and directed them to give it at short intervals, so that he should take it all by the middle of the day, at which time, if it should not have operated *freely*, to give another portion of calomel, combined with jalap, and afterwards to give a small dose of castor oil, every two hours, till it *did operate*.

On Tuesday, 31st, again visited my patient. Had taken all the medicine, without its producing any operation; found him very restless and uneasy; the tumefaction of the abdomen was rather greater than before; complained of much griping pain; started and moaned in his sleep; in short all the symptoms were rather aggravated. I made use of a strong solution of sulphate of soda, in a decoction of senna, as an enema, throwing up between half a pint and a pint at a time; this was discharged in about half an hour after each administration, without producing any other evacuation, than a few worms, with what was thrown up. I directed a small portion of calomel, scammony, and jalap, to be given three or four times in the day, combined with a carminative, to prevent the griping, as well as its rejection by vomiting; the enemata were to be continued at intervals of three or four hours, and warm emollient fomentations applied to the abdomen.

Wednesday, 1st April. No evacuation, and what was thrown in by injection remained much longer than heretofore. The patient was evidently worse, was much debilitated, with great anxiety of countenance. The breathing was hurried and laborious; pulse frequent, small and feeble; tongue white, dry, and sticky; breath extremely fetid; and the tumefaction and soreness of the abdomen much increased. I directed the enemata to be continued, with an addition of ten or twelve grains of tartarized antimony, to each portion thrown up; a powder composed of 4 grs. of calomel, 4 of scammony, and 5 of jalap, to be given every two hours until eight were taken; then to give a small portion of castor oil at the same intervals, and, besides, to drink of a decoction of spigelia and senna, through the day. A large blister was laid over the front part of the abdomen, in order to allay, as much as possible, the irritation within, and to prevent the inflammation which, from the extreme soreness, I feared might take place.

Thursday, April 2d. Not much alteration since yesterday. The blister had drawn well, and he had been more composed, had slept quietly for several hours, as soon as the blister had drawn. The enemata had no better effect than before. I directed the same course to be continued, the same number of the cathartic powders to be given, as yesterday, and the castor oil afterwards. Increased the quantity of tartarized antimony in each injection to 15 or eighteen grains, and the patient to be put in the warm bath two or three times in the course of the day.

Friday, 3d. No alteration except for the worse. The injections, notwithstanding the increased quantity of tart. ant. had remained much longer than heretofore; they would occasionally bring away a worm or two, without producing any other cathartic effect. The little patient appeared to be fast sinking into the arms of death; a very feeble and frequent pulse, listlessness and insensibility, great debility, tongue moist, white, and slimy, considerably comatose, distension of the abdomen about the same, but the soreness was less than before the blister was applied. I directed a pretty free use of brandy or wine, mixed with water. Made a trial of the tobacco injection; it was discharged immediately, without bringing away any thing more than was thrown up. I began to despair of effecting any thing by the use of injections, or, indeed, of strong cathartic medicines, as there had already been so much taken without producing any evacuation. I however directed the warm bath, and in consequence of his debility, I had it applied by means of a blanket wrung out of hot water; the patient being stripped and wrapped in it, as warm as he could conveniently bear it. I also advised a pretty free use of wine and water, and a small dose of castor oil to be taken every two, three, or four hours.

Saturday, 4th. I was much gratified on visiting my patient, to find that the medicine had began to operate. The first motion from its bowels brought away a convoluted knot or roll of worms, which consisted of seventy in number, mixed with a large quantity of dark coloured, slimy, feculent matter, of a very disagreeable fetid odour. The medicine continued to operate throughout the day, and, indeed, for several days. The stools were pretty much of the same nature, and mixed with the same kind of worms, viz. *lumbricoides*, from four to eight or nine inches in length. They were nearly or quite all dead, and many of them pretty far advanced towards a state of putrefaction, indicating that they had been dead for several days. The number discharged was so great, as to induce the parents to count them. In the three first days, the number discharged was about four hundred, and during the week five hundred and fifty-two, all of the above size.

Almost as soon as the medicine operated, the child had a good appetite, which it was found necessary rather to restrain than encourage. It advanced rapidly in strength, and was in a short time restored to its former health and spirits.

On reflection upon the case, I am led to the conclusion, that a principal reason of the obstinate constipation, was merely a mechanical obstruction of the intestines, by the knots or rolls of worms which they contained; for as soon as the first large roll was discharged, (which was of itself quite sufficient, completely to obstruct any part of the alimentary canal,) the cathartic effect of the medicine appeared to go on very naturally. The indurations, which could be felt in the abdomen along the course of the colon, I have no doubt were of this nature, from the circumstance of their frequently changing their positions, and entirely disappearing immediately on the operation of the cathartic medicine.

The whole amount of medicine which the patient took during the week, before it operated, was about 100 grs. calomel, 75 of scammony, 75 of jalap, and 2 pints of decoction of spigelia and senna, besides a large quantity of oil and epsom salts, given by the mouth, and an incredible quantity of senna, salts, and tartarized antimony by injections.

*June*, 1829.

